# Impact of microalgae layer thickness on the treatment performance of drain water

**DOI:** 10.1038/s41598-023-48129-x

**Published:** 2023-11-27

**Authors:** Aya Moustafa Moustafa, Mohamed El-Hosseiny ElNadi, Mahmoud Mohamed Abdelmomen, Amira Mohamed Nagy

**Affiliations:** Sanitary Engineering Section, Public Works Department, Faculty of Engineering, Ain Shams University, Cairo, Egypt

**Keywords:** Civil engineering, Environmental impact

## Abstract

The water shortage problem in Egypt has promoted the exploration of new water resources, including the use of treated agricultural drainage water. This study aims to develop an efficient and cost-effective method for the in-situ treatment of agricultural drainage water from the Bahr-ElBaqar drain using a microalgae layer. The objective was to establish the optimal thickness of the layer for achieving the highest removal efficiency of pollutants from the drain's wastewater. Practical work was performed on a pilot consisting of five channels with four channels having microalgae with different thicknesses and fixed lengths of 50 cm, and the fifth channel acting as a buffer channel to assimilate the drain water without any treatment microalgae layer. After the experiment, it was discovered that a 10-mm layer of microalgae was the most effective thickness for eliminating pollutants from wastewater. The removal efficiencies were 29% for biochemical oxygen demand (BOD), 46.9% for chemical oxygen demand (COD), and 56.1% for total suspended solids (TSS) removal. This experiment provided evidence that microalgae could represent a viable solution for in-situ treatment of agricultural drainage wastewater with high removal efficiencies for pollutants in wastewater and decreased the need for constructing huge and expensive wastewater treatment plants.

## Introduction

The water scarcity problem in Egypt is widely increasing due to the increasing population, increasing standard of living, urban, and industrial activities. This led to an increase in the gap between water demand and available water resources. Therefore, the need for non-conventional water resources appeared^1^. Conventional water resources such as the Nile River, Groundwater, and Rain precipitation while unconventional water resources as using treated municipal and agricultural drainage water, desalination, and groundwater in the delta region. Conventional water resources such as the Nile River, deep groundwater, and rain precipitation could afford about 62.65 BCM/year; 55.5 BCM by the Nile River, 6.1 BCM by groundwater, and 1.05 BCM by the rain precipitation. While the annual demand for water is about 79.5 BCM. The non-conventional resources such as using treated municipal wastewater, treated agricultural drainage water, and desalination could afford about 12.7 BCM; 9.7 BCM by using treated agricultural drainage water, 2.9 BCM by using treated municipal wastewater, and 0.1 BCM by desalination^2^. It’s obvious that the annual amount of agricultural drainage water is huge which will require a high cost of buildings, operation, and maintenance of WWTP for the treatment of such a huge amount with the conventional methods, as a result, means must be explored for the treatment of agricultural drainage water with low cost and high efficiency to get the benefit of using this treated wastewater^3^.

The strategy of agricultural drainage water reuse is considered one of the most important uonventional water resources and it can contribute to overcoming the shortage of water problem in Egypt, saving fresh water, and improving the Physicochemical properties of soil. It’s an economical and effective method that could produce high-quality water instead of more expensive methods such as municipal wastewater treatment or seawater desalination^2^.

A strong open drainage system was constructed in Egypt along the Nile to transfer excess water to the Mediterranean Sea and lakes^4^. It has been used since 1970 in Lower Egypt^5^. Agriculture drains were originally made to collect irrigation drainage but due to increasing population, and seepage of sewerage facilities, these drains now carry different types of polluted water because untreated industrial wastewater is discharged into them similarly to untreated domestic wastewater, also, the absence of sanitation systems in rural areas drives the farmers to discharge their wastewater into agricultural drains^6^. As a result, these drains became fully loaded with different types of pollutants from agricultural, industrial, and domestic wastewater. This lead to several adverse impacts such as depletion of dissolved oxygen level in drains, which may affect the aquatic life in drains, also, self-purification ability will decrease. As a result, the direct reuse of water from agricultural drains becomes very risky and requires treatment for the drains’ water to improve its quality for reuse purposes or to conform to regulatory disposal requirements^7,8^.

Several scenarios in agricultural water treatment can be used according to treatment procedure and location. The current study will focus on the treatment inside the stream body itself, in this method the whole volume of the drain is treated inside the stream body to enhance the level of water quality to simulate and accelerate the self-purification effect^9^. In-situ treatment has many advantages compared to other techniques, such as low costs, less adverse impacts on the environment, and no secondary production of pollutants^10^. Several methods of In-situ treatment could be applied inside the stream. All of them depend on the biodegradation process for the removal of pollutants. These methods are classified according to treatment procedure to Hydraulically Method, Mechanical Method, and Biological Method. This research will focus on the biological method as follows.

### Biological method

In this method, biodegradable materials can be used to complete the degradation process in cooperation with bacteria. These materials may be agricultural wastes, plastic media^11^ or maybe through the bioremediation process which uses living organisms in completing the biological action and degradation of organic matter, heavy metals, and toxics such as (aquatic weeds, floating plants, algae, fungi, yeasts, and bacteria) with minimum construction, operation, and maintenance cost^12^.

Using microalgae in wastewater treatment shows promising results. Because they can deal with various types of pollutants in wastewater. This owes to their metabolic flexibility and their ability to do photoautotrophic, mixotrophic, or heterotrophic metabolism^13^. The role of microalgae in wastewater treatment is the direct uptake of pollutants through adsorption or ion exchange or transformation of pollutants, and providing oxygen to the bacteria to help bacteria in the aerobic degradation of the pollutants in water which decreases the cost of artificial aeration compared to the other wastewater treatment techniques^14^. This gave it many advantages over other ordinary physiochemical techniques such as (reverse osmosis, electro-dialysis, membrane separation, activated carbon adsorption, and chemical oxidation or reduction, etc.). These advantages are low operational cost, removal of bacteria and heavy metals, consumption of CO_2_ from the atmosphere, production of homogenous biomass, and production of high-value products and biomolecules^15^ removal of organic compounds, oxygenating effluent before releasing it into a water body, eliminating the requirement for sludge handling, and using biomass as fertilizer after removing N, P which is considered an environment-friendly process^16,17^.

### Microalgae treatment methodology inside stream body

Microalgae can absorb and enrich heavy metals, plant nutrients, organic and inorganic pollutants, pesticides, and radioactive substances in their unicellular bodies. In particular, improving water quality has benefited from this capacity to reduce Nitrogen, Phosphorus, and Heavy metals (HM), and it offers a more straightforward, practical, and affordable alternative to conventional environmental cleanup techniques^18^. Physical, biological, and biochemical techniques used to minimize pollutants including inorganic and organic pollutants are described as follows:

#### Bioadsorption

Bioadsorption is the process of compounds being adsorbed passively onto the cell wall or extracellular polysaccharides (EPS) excreted by microalgae. There are two types of EPS: one is attached to the cell wall, and the other is free and secreted into the growth medium, both of which can be utilized to adsorb pollutants in wastewater. The effectiveness of bio-adsorption is influenced by the characteristics of the pollutants, including their structure, functional groups, hydrophobicity, and the type of microalgae used. The negatively charged functional groups, such as carboxyl, hydroxyl, and phosphoryl, present in microalgae and EPS, attract positively charged groups. Bio-adsorption involves various mechanisms, such as ion exchange, complex reactions with the surface, micro-precipitation, adsorption reactions, and chelation^19^.

#### Bioaccumulation

Bioaccumulation is described by the bio-concentration factor (BCF) which represents the ratio of a specific contaminant's concentration within an organism to its concentration in the surrounding environment. Various factors influence the BCF levels, including the availability of chemicals, metabolism, bioconcentration process, physical barriers, ionization of ionizable molecules, dissolved organic matter, BCF testing techniques, and the surrounding environment^20^.

#### Intracellular degradation

During bio-uptake, the contaminant penetrates the cell through the cell wall and binds to intracellular proteins and other substances. Unlike adsorption, bio-uptake of pollutants into the cell occurs over hours to days and is only observed in living microalgal cells. The three primary mechanisms by which emerging contaminants are absorbed across the cell membrane by microalgae are passive diffusion, passive-facilitated diffusion, and energy-dependent/active uptake^17^.

Varied microalgae strains have different characteristics, which would also affect how well they can remediate wastewater. Therefore, the wastewater properties, the effluent treatment level, the cost and energy need for biomass harvesting, and the application of the harvested biomass would be the most important factors to take into account when picking a strain or mix-consortia for wastewater treatment^21^.

In 2015, Nurfarahana et al. made an experiment using chlorella species in the bioremediation of aquaculture wastewater. The experiment showed good results in the removal of nutrients. They reported a correlation between the growth kinetics of Chlorella sp. and the nutrients N-NH_4_^+^ and P-PO_4_^-^ contained in aquaculture wastewater; indicating that the optimal inoculation of Chlorella was 30% (v/v), by which the removal of N-NH_4_^+^ and P-PO_4_^−^ was 98.5 and 92.2 4%, respectively^22^.

In 2018, One Water Company created the algae wheel system, which is a modern algal-fixed film technology. The biofilm ecosystem linked to the algae wheels consists of a mix of algae and bacteria, and the synergistic action of both types of microorganisms improves the whole system's treatment efficiency. Microalgae use sunlight to take in the CO_2_ that bacteria emit. Photosynthesis produces polysaccharides, which serve as both a bacterial feed supply and a solid settler. In turn, the bacteria may use photosynthetically generated oxygen, producing a self-regulating and ecological WWT system that is stable^13^.

A project in Latin America evaluates the ability of two strains of microalgae (*Chlorella* and *Scenedesmus sp.*) and one cyanobacteria to remove excess pesticides and other nutrients present in runoff water from rice production. Different concentrations of wastewater were evaluated. According to the results, the two strains significantly reduced the concentration of NO_3_ and PO_4_ (95 and 85%, respectively). Also, *Chlorella sp*. obtained the highest removal efficiency of the pesticide (Chlorpyrifos), followed by Scenedesmus sp. (100%, and 75% respectively)^15^.

In 2020, a study was carried out in Manzala Lake using a pilot-scale wetland consisting of four parallel basins exposed to natural climatic conditions. The first basin was cultivated with reeds, while three different microalgae species (*chlorella, spirulina, and Azolla*) were cultivated in the three other basins. They experimented with the removal of different contaminants such as BOD, COD, TN, TP, TC, FC, and heavy metals. Results showed that BOD and COD best removal was reached by chlorella with 88 and 84% removal efficiencies respectively. *Spirulina* was found to be the most efficient in extracting heavy metals such as aluminum, iron, and manganese by 89, 97, and 86% respectively^23^.

The primary objective of this study was to identify an economically viable approach for the insitu treatment of agricultural drainage water using microalgae. The study aimed to determine the optimal thickness of the microalgae layer required for effectively treating agricultural drainage water from the Bahr El-Baqar drain, also the study aimed to develop a treatment approach that is both economically viable and easy to implement, operate, and maintain, while ensuring optimal treatment efficiency.

## Materials and methods

**Figure 1:**
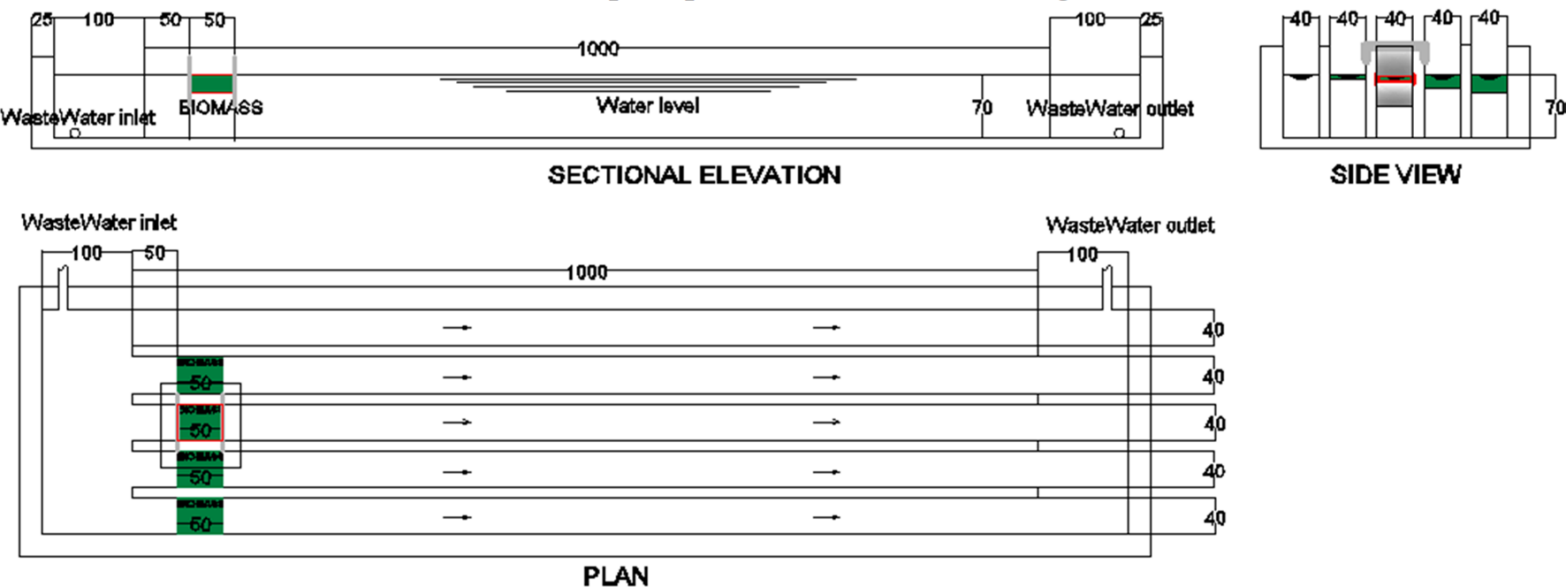
Pilot unit detailed drawing.

The experimental work was done in a pilot that was operated under the natural conditions of temperature, sunlight, and humidity according to the study period. The pilot was built from several parts including the main parallel five channels that simulate the drain. These channels were of dimensions 40 cm in width, 70 cm in depth, and length of 10 m. They were fed and drained by two crossing channels one at the beginning and the second at the end of the five channels each with 70 cm depth and 1 m width and have a feeding pipe of diameter 4-inch from a submersible pump of 10 l/s and head 10 m erected inside the drain, and a disposal pipe of diameter 6-inch at its ends to return water to the drain. The flow of water from the drain to the pilot was 10 l/s, with 0.035 m/s velocity inside the channels of the pilot, and 14 s hydraulic retention time inside the microalgae layer. The applied biomass as a treatment procedure was placed in each channel 50 cm from its beginning with a length of 50 cm that was fixed by stainless steel plates (that are fixed by U-shaped handles at the walls of the channels, and extend from the top of the channel’s wall to 15 cm below the microalgae layer), and different average thicknesses of (5, 10, 15, and 20 mm) for the biomass layer; microalgae of thicknesses (2–7.5 mm) was used to give an average thickness of 5 mm, etc., Plan and sectional elevation, and side view for the pilot plant unit are shown in Fig. 1.

The operation procedures for the practical work are discussed as follows.

- After pilot construction and preparation, the submersible pump was operated to deliver raw wastewater from the drain to the pilot.
- The outlet valve was closed till the pilot was filled with water and then opened for continuous flow.
- Before starting the treatment run one preparation week was made to ensure the biomass stability with the flow, and the continuity of operation.
- After one-week preparation, the main run was made for 6 weeks in its operation as follows:
  - Several microalgae layer thicknesses between 5 and 20 mm on average were used during the operation period, all with a length of 50 cm, one in each channel, leaving channel no. 1 without a microalgae layer as a buffer.
  - Samples were taken from the inlet crossing channel, after 0.5 m from the microalgae layer, from the middle of the five longitudinal channels, and at the disposing crossing channel, The samples were taken from each location three times per day at 9.00 am, 1.00 pm, and 6.00 pm to simulate the variation during the day then the average of the three readings was taken to represent the value of the measured parameter in the day. This was done for 6 weeks, for six working days per week during the study period.
  - The measured parameters were BOD, COD, and TSS.
    - **BOD** is usually expressed as the amount of oxygen used by the aerobic bacteria to oxidize the biodegradable organic matter at temperature 20 C° for 5 days under aerobic conditions. It was measured by BOD measuring instrument according to the standard method for examination of water and wastewater^24^. Measuring BOD helps to assess organic pollution; High BOD levels indicate the presence of large amounts of organic matter, such as agricultural residues, fertilizers, and animal wastes in the wastewater so this would help in quantifying the organic pollution load, Determine treatment requirements, and monitor the effectiveness of treatment processes in removing these pollutants, and ensure compliance with environmental regulations. In the context of the treatment efficiency; High BOD levels indicate a higher concentration of organic pollutants, which can deplete oxygen levels in water bodies when discharged untreated. This oxygen depletion can harm aquatic life, inhibit the oxidation of organic matter by aerobic bacteria which slow the self-purification process and disrupt the ecological balance.
    - **COD** is the amount of oxygen used to oxidize all organic compounds present in wastewater chemically whether they are biodegradable or non-biodegradable using a strong oxidizing agent for three hours under temperature 200C°. It was measured according to the standard method for examination of water and wastewater^25^, Measuring COD helps to Comprehensive organic characterization: Unlike BOD, COD includes both biodegradable and non-biodegradable organic compounds. This measurement provides a more comprehensive assessment of the organic matters load, including substances that may be non-biodegradable by bacteria. It helps in understanding the overall treatment requirements, Treatment process evaluation, and ensure compliance with environmental regulations. In the context of the treatment efficiency; High COD levels in discharged wastewater can lead to oxygen depletion in water bodies, harming aquatic life and disrupting ecosystems. It causes poor water quality, including odors and discoloration. It can also contribute to harmful algal blooms and pose risks to human health if the wastewater is used for irrigation or drinking water sources downstream.
    - **TSS** refers to waterborne particles that exceed 2 microns in size. The majority of total suspended solids comprise of inorganic materials; however, algae and bacteria may also be considered. They were measured according to the standard method for examination of water and wastewater using filter paper, a Filtration apparatus, and a Suction flask to filter the sample through it. Filter paper was first dried and weighed using a sensitive analytical balance, then the water sample was filtered using a filter paper suction flask and filtration apparatus, the filter paper was dried in the drying oven under temperature 103 to 105°C for one hour, left to cool in a glass desiccator until they reach room temperature then weighed using the analytical digital balance and the difference between the weight of the empty filter paper and its weight after drying represents the TSS^26^. Measuring TSS helps to Indicate solids content including sediment, organic matter, and particulate pollutants. Evaluation of treatment efficiency in removing solid particles. In the context of the treatment efficiency Monitoring TSS levels before and after treatment, would help in determining the efficiency of sedimentation, filtration process, Protection of downstream ecosystems: High TSS levels can negatively impact aquatic ecosystems by reducing light penetration, increasing turbidity, and affecting the integrity of habitats. So measuring and controlling TSS helps prevent these adverse effects and protect downstream environments.

### Consent to participate

All authors consent to participate in the research project.

## Results and discussion

The average results of the daily measurements for the mentioned parameters and the discussion of the results are presented as follows.

### BOD concentration daily results

**Figure 2:**
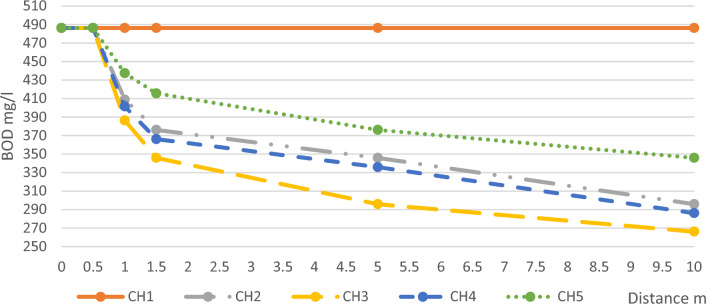
Average BOD Concentrations along the Five Channels.

The average values of BOD daily measurements and the removal ratios of BOD compared with channel 1 are illustrated in Table 1 and Fig. 2.

**Table 1:** Average BOD results and removal ratios.

Distance m	CH1		CH2		CH3		CH4		CH5	
	Conc. mg/l	R.R%	Conc. mg/l	R.R%	Conc. mg/l	R.R%	Conc. mg/l	R.R%	Conc. mg/l	R.R%
0	486	0	486	0	486	0	486	0	486	0
1.5	486	0	376	23	346	29	366	25	416	15
5	486	0	346	29	296	39	336	31	376	23
10	486	0	296	39	266	45	286	41	346	29

According to the short length of the pilot’s channel which is 10 m, the concentration of BOD in the buffer channel remained almost constant along the channel length^27^. This is due to the low concentration of dissolved oxygen (DO) inside the channel which limits bacterial activity with organic matter. It needs a very long distance to make the bacterial action take place as self-purification because of the increase of DO with water velocity and air friction^28^.

After the microalgae layer, the removal ratio was affected by the layer thickness varied between (15 to 29%) with an optimum value at the layer of thickness 10 mm, and this agreed with the study that proved the success of microalgae in the removal of 51% of BOD from wastewater^29^, and in another study by ratio 93%^13^ (but here we are discussing the effect of thickness of microalgae layer on the removal efficiency of microalgae), also the BOD removal continued inside the channel due to the availability of enough DO for oxidation needs. However, the removal rate decreased over time. After the microalgae layer, high DO was left which makes the degradation action highly work between bacteria and organics^30^. Then, at the middle of the channel and the end of it, the water velocity can’t recover the consumed DO which leads to the degradation weakness^28^. After the decrease of DO concentration to be the same as the influent, the bacterial degradation will return to be very slow as happened in the buffer channel.

According to thickness, the removal of BOD increased as the thickness of the Microalgae layer increased. It was between ranges (23–39%) in channel 2 compared with raw water and increased within ranges (29–45%) in channel 3 along the different measurement distances. But it decreased within ranges (25–41%) in channel 4 and decreased more in channel 5 as it was within ranges (15–29%).

**Figure 3:**
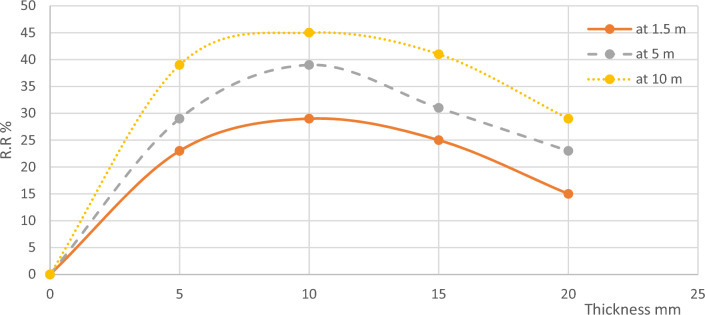
BOD Removal Ratios According to Thickness.

From the previous results, the effect of microalgae layer thickness on BOD removal showed a bell-shaped curve with an average thickness of 10 mm on the optimum value as shown in Fig. 3 this is because the photosynthetic activity of the microalgae works up to a total layer thickness of 10 mm, beyond this thickness the lower part doesn’t work with photosynthesis but consumes oxygen as normal plant activity^31^.

### COD concentration daily results

**Figure 4:**
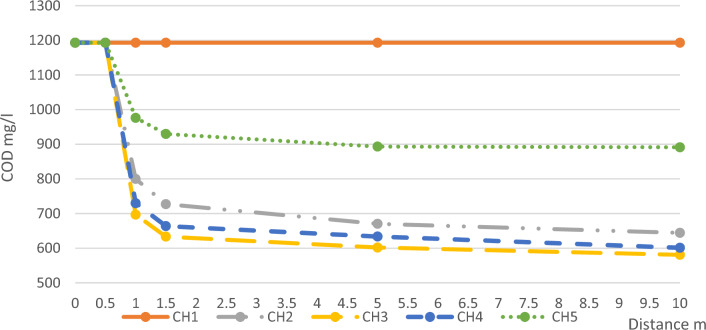
Average COD Concentrations along the Five Channels.

The average values of COD daily measurements and the removal percentages of COD in each channel compared with channel 1 are illustrated in Table 2 and Fig. 4.

**Table 2:** Average COD Results and Removal Ratios.

Distance m	CH1		CH2		CH3		CH4		CH5	
	Conc. mg/l	R.R%	Conc. mg/l	R.R%	Conc. mg/l	R.R%	Conc. mg/l	R.R%	Conc. mg/l	R.R%
0	1193	0	1193	0	1193	0	1193	0	1193	0
1.5	1193	0	727	39	633.3	46.9	663.61	44.4	929.72	22.1
5	1193	0	670.29	43.8	602	49.5	633.61	46.9	893.33	25.1
10	1193	0	644.29	46	580.8	51.3	601.11	49.6	891.38	25.3

COD concentration almost remained constant in the buffer channel which can be attributed to the insufficient availability of DO necessary to initiate oxidation processes. This shortage of DO concentration can be attributed to the relatively short length of the pilot channel, measuring 10 m^27^, which hindered the establishment of optimal conditions for self-purification. Self-purification primarily relies on the enhancement of DO concentrations in water, increased by water velocity, and frictional interactions with atmospheric air^28^. These factors contribute to an increased DO level, promoting oxidation reactions essential for the successful removal of COD. However, due to the limited length of the channel, the desired conditions for effective self-purification and subsequent COD removal were not adequately established.

After the microalgae layer, the removal ratio was noticeable and affected by the layer thickness, it varied between (22.1 to 46.9%) with an optimum value at the layer of thickness 10 mm and this agreed with the study that proved the success of microalgae in the removal of 91% of COD from wastewater^29^ and in another study by 95%^32^. Also, the COD removal continued inside the channel after the microalgae layer due to the availability of enough DO for oxidation of organic and inorganic matters that increased the removal ratios of COD but with declining rates. This is because near the microalgae layer the produced oxygen by microalgae is high which increases the DO concentration in water, this helped in the oxidation of both organic substances (by bacteria) and inorganic substances. But as the distance increased from the microalgae layer the concentration of DO decrease which decreased the oxidation rate until it became very slow (linear) as the buffer channel where the DO concentration is as influent^30^.

According to thickness, COD removal efficiency was different in the four channels according to the thickness of the microalgae layer. In channel 2, the removal efficiencies were within the range of (39–46%) but it increased in channel 3 to the range of (46.9–51.3%), while it decreased as the thickness of microalgae increased, It decreased in channel 4 to (44.4–49.6%) and in channel 5 to (22.1–25.3%) in the different sampling locations along the length of the channels.

**Figure 5:**
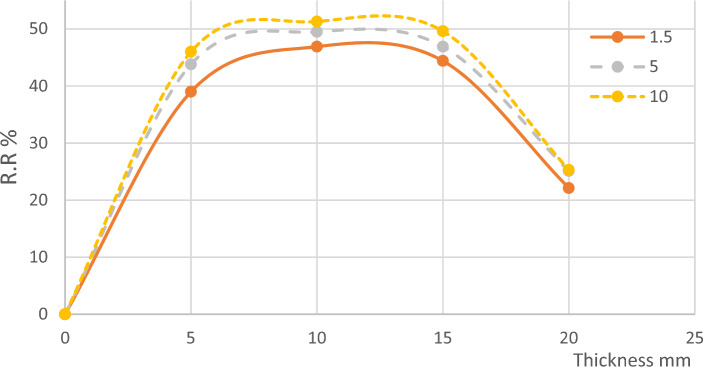
COD Removal Ratios According to Thickness.

From the previous results, the effect of microalgae layer thickness on COD removal took the bell shape with an average thickness of 10 mm on the optimum value as shown in Fig. 5 as it was the best thickness that achieved the best sunlight feed to the microalgae layer which produced the highest DO that is the main factor in the oxidation process. But as the thickness increased as in channels 4 and 5 the lower part didn’t receive the sunlight that prevented its photosynthetic activity, while if the layer thickness is less, the removal efficiency also decreased due to the incomplete microalgae layer^31^.

### TSS concentration daily results

**Figure 6:**
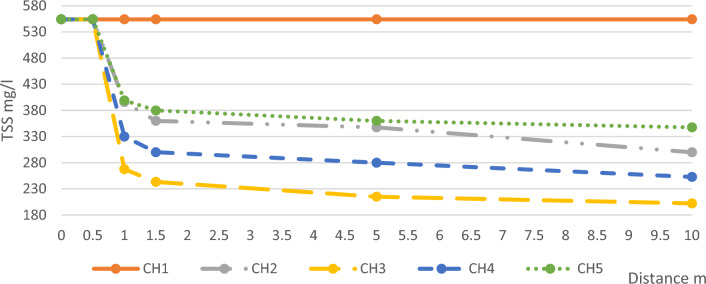
Average TSS Concentrations along the Five Channels.

The average values of TSS daily measurements, and the removal percentages of TSS in each channel compared with channel 1 are illustrated in Table 3 and Fig. 6.

**Table 3:** Average TSS Results and Removal Ratios.

Distance m	CH1		CH2		CH3		CH4		CH5	
	Conc. mg/l	R.R%	Conc. mg/l	R.R%	Conc. mg/l	R.R%	Conc. mg/l	R.R%	Conc. mg/l	R.R%
0	554	0	554	0	554	0	554	0	554	0
1.5	554	0	360	35	243	56.1	300	45.9	380	31.4
5	554	0	348	37.3	215	61.2	280	49.5	360	35
10	554	0	300	45.9	202	63.5	253	54.4	3478	37.3

TSS removal efficiency depends on the oxidation reaction that resulted in settlable substances, as the rate of oxidation action increase this will result in more settleable substance that settles under the effect of gravity in a hindered settling way that sweeps and block small, suspended solids and force them to settle down, also TSS removal efficiency can be attributed to the capacity of the microalgae layer to attract the suspended particles in the water and adhere them to its surface. This is possible because the microalgae layer possesses a strong ability to absorb and retain these solids, leading to effective removal^33^.

TSS removal in the buffer channel almost remained constant as no oxidation process happened due to the insufficiency of DO concentration so no settleable matters were formed and due to the short distance of the pilot’s channel the time for precipitating the existing suspended solids was too short. Furthermore, the absence of an adhering microalgae layer that could adsorb TSS on its surface also contributed to this phenomenon^27–33^.

After the microalgae layer, the removal ratios were high, they were affected by the layer thickness and varied between (31.4–56.1%) with an optimum value at the layer of thickness 10 mm. TSS removal continued inside the channel away from the microalgae layer but with declining rates because the remaining DO concentration continued to decrease and that was obvious in decreasing the rate of BOD and COD removal along the length of the channels resulted in the precipitation of less produced settleable matter.

According to the thickness, the removal efficiency of TSS increased in channel 2 within ranges (35–45.9%) and it increased in channel 3 to ranges (56.1–63.5%), But the T.S.S removal efficiency decreased in channel 4 to ranges (45.9–54.4%) and in channel 5 to (31.4–37.3%).

**Figure 7:**
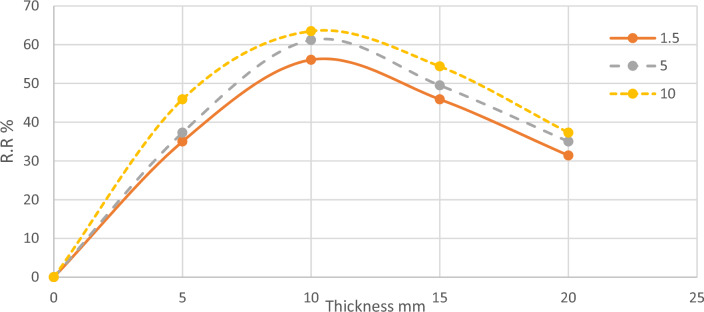
TSS Removal Ratios According to Thickness.

From the previous results, it was observed that the thickness of the microalgae layer had a significant impact on TSS removal, following a bell-shaped curve with the optimum value being around 10 mm, as shown in Fig. 7. In channel 3, where the microalgae layer had an average thickness of 10 mm, there was an increased bacterial degradation activity for organic matter and oxidation of inorganic matter. This resulted in the production of more settleable materials that could settle under the effect of gravity, leading to a higher efficiency of TSS removal. However, when the microalgae layer thickness exceeded this optimum value, a different phenomenon occurred. The lower part of the microalgae layer began to resemble a typical plant and lacked the necessary access to sunlight required for the photosynthesis process. As a consequence, the oxidation reaction decreased, resulting in a reduced production of settleable materials. Consequently, the removal efficiency of TSS also decreased. Furthermore, it should be noted that the lower part of the microalgae layer itself acted as a dead zone, contributing to an increase in TSS within the wastewater. While if the microalgae layer is less, the TSS removal efficiency also decreased due to the uncomplete microalgae layer.

## Conclusion

The primary objective of this scientific research was to determine the optimal thickness of a microalgae layer for in-situ treatment of agricultural drainage water. To achieve this, a pilot study was conducted using five channels, with four channels equipped with microalgae layers of varying thicknesses (ranging from 5 to 20 mm) and length 50 cm, while one channel served as a buffer to simulate natural drainage conditions.

The results of the experiment, as discussed in the findings, clearly demonstrate the effectiveness of microalgae as a treatment technique for agricultural drainage wastewater, exhibiting promising initial removal efficiencies. However, it was observed that the removal efficiency depended on the thickness of the microalgae layer. Initially, there was an increase in the removal of the targeted parameters (BOD, COD, and TSS) as the thickness increased from 5 to 10 mm. Nevertheless, it is important to note that this trend did not persist, as the removal efficiency started to decline beyond a thickness of 10 mm, reaching its lowest point at a thickness of 20 mm.

Remarkably, the channel featuring a microalgae layer with an average thickness of 10 mm exhibited the highest removal efficiency for BOD, COD, and TSS, with removal ratios of 29%, 46.9%, and 56.1%, respectively.

In conclusion, this study underscores the potential of microalgae as a viable treatment technique for agricultural drainage wastewater, with an emphasis on the critical role played by the thickness of the microalgae layer. By identifying the optimal thickness of 10 mm, the research provides valuable insights for the effective removal of BOD, COD, and TSS, thereby contributing to the advancement of sustainable agricultural wastewater treatment practices.

## Data Availability

Enquiries about data availability should be directed to the authors.

## References

[1] Ashour MA, El Attar ST, Rafaat YM, Mohamed MN (2009). Water resources management in Egypt. J. Eng. Sci. Assiut Univ..

[2] AbdEllah RG (2020). Water resources in Egypt and their challenges, Lake Nasser case study. Egypt. J. Aquatic Res..

[3] Ashour E, Zeidan B, Elshemy M (2021). Assessment of agricultural drainage water reuse for irrigation in El-Behira Governorate, Egypt. J. Water Sci..

[4] MWRI, ‘‘National Water Resources Plan for Egypt 2017’’. http://extwprlegs1.fao.org/docs/pdf/egy147082.pdf.

[5] Zidan, M., S., & Dawoud, M., ‘‘Agriculture Use of Marginal Water in Egypt: Opportunities and Challenges’’, Developments in Soil Salinity Assessment and Reclamation, (2013).

[6] Hassanain N, Shaapan R, Saber M, Kabary H, Zaghloul A (2021). Adverse impacts of water pollution from agriculture (crops, livestock, and aquaculture) on human health, environment, and economic activities. Egypt. J. Aquatic Biol. Fish..

[7] Abd-Elhamid, H., El-Gohary, E., Elnag, Z.A., ‘‘Safe reuse of treated wastewater for agriculture in Egypt’’, Conference: EXCEED-SWINDON EXPERT WORKSHOP, Water Efficient Cities", Morocco, November 2017.

[8] Khalil, E., M., M., ‘‘Enhancing Agriculture Drainage Water Quality to Improve Water Use Efficiency in Middle Delta Region’’, MWRI_Central Library, Egypt, 2016.

[9] Fleifle A, Allam A, Negm A (2016). Remediation of agricultural drainage water for sustainable reuse. The Nile Delta.

[10] Ateia M, Yoshimura C, Nasr M (2016). In-situ biological water treatment technologies for environmental remediation: A review. J. Bioremediat. Biodegrad..

[11] Ghazy M, Basiouny M, Badawy M (2016). Performance of agricultural wastes as a biofilter media for low-cost wastewater treatment technology. Adv. Res..

[12] Saeed MU, Hussain N, Sumrin A, Shahbaz A, Noor S, Bilal M, Aleya L, Iqbal HMN (2022). Microbial bioremediation strategies with wastewater treatment potentialities—A review. Sci. Total Environ..

[13] Wollmann F, Dietze S, Ackermann J, Bley T, Walther T, Steingroewer J, Krujatz F (2019). Microalgae wastewater treatment: Biological and technological approaches. Eng. Life Sci..

[14] Abdel-Raouf N, Al-Homaidan AA, Ibraheem IBM (2012). Microalgae and wastewater treatment. Saudi J. Biol. Sci..

[15] Castellanos-Estupiñan MA, Carrillo-Botello AM, Rozo-Granados LS, Becerra-Moreno D, García-Martínez JB, Urbina-Suarez NA, López Barrera GL, Barajas-Solano AF, Bryan SJ, Zuorro A (2022). Removal of nutrients and pesticides from agricultural runoff using microalgae and cyanobacteria. Water.

[16] Koul K, Sharma K, Shah MP (2022). Phycoremediation: A sustainable alternative in wastewater treatment (WWT) regime. Environ. Technol. Innov..

[17] Sutherland DL, Ralph PJ (2019). ‘Microalgal bioremediation of emerging contaminants—Opportunities and challenges’. Water Res..

[18] El Helw EAA (2015). Ecological Sustainability and Biological Treatment for Agricultural Drainage Water Polluted with Pesticides from Different Delta Fields.

[19] Al-Jabri H, Das P, Khan S, Thaher M, AbdulQuadir M (2021). Treatment of wastewaters by microalgae and the potential applications of the produced biomass—A review. Water.

[20] El-Sheekh M, El-Dalatony MM, Thakur N (2022). Role of microalgae and cyanobacteria in wastewater treatment: Genetic engineering and omics approaches. Int. J. Environ. Sci. Technol..

[21] Plöhn M, Spain O, Sirin S, Silva M, Escudero-Oñate C, Ferrando-Climent L (2021). Wastewater treatment by microalgae. Physiologia Plantarum.

[22] Mohd NN, Abu BNS, Lananan F, Abdul HSH, Lam SS, Jusoh A (2015). Treatment of African catfish, Clarias gariepinus wastewater utilizing phytoremediation of microalgae, Chlorella sp. with Aspergillus niger bioharvesting. Bioresour. Technol..

[23] Agamy S, El Saadi A, Galal MM (2020). Application of algae to free surface wetlands for effluent reuse. Water Environ. J..

[24] Standard Methods Committee of the American Public Health Association, American Water Works Association, and Water Environment Federation. 5210 biochemical oxygen demand (bod) In: “Standard Methods for the Examination of Water and Wastewater”. Lipps WC, Baxter TE, Braun-Howland E, editors. Washington DC: APHA Press. DOI: 10.2105/SMWW.2882.102

[25] Standard Methods Committee of the American Public Health Association, American Water Works Association, and Water Environment Federation. 5220 chemical oxygen demand (cod) In: “Standard Methods for the Examination of Water and Wastewater”. Lipps WC, Baxter TE, Braun-Howland E, editors. Washington DC: APHA Press. DOI: 10.2105/SMWW.2882.103

[26] APHA-AWWA-WPCF, ‘‘Standard Methods for the Examination of Water and Wastewater’’, American Public Health Association, American Water Works Association, Water Environment Federation, American Public Health Association Publishment, 19th edition WDC, USA, 1999.

[27] Šaulys V, Survilė O, Stankevičienė R (2020). An assessment of self-purification in streams. Water.

[28] kumar, R., ‘‘Self-Purification of Natural Streams | Sewage Treatment |Waste Management’’ Environmental pollution. https://www.environmentalpollution.in/sewage-treatment/self-purification-of-natural-streams-sewage-treatment-waste-management/5310.

[29] Marella TK, Parine NR, Tiwari A (2018). Potential of diatom consortium developed by nutrient enrichment for biodiesel production and simultaneous nutrient removal from waste water. Saudi J. Biol. Sci..

[30] Novoveská L, Zapata AKM, Zabolotney JB, Atwood MC, Sundstrom ER (2016). Optimizing microalgae cultivation and wastewater treatment in large-scale offshore photobioreactors’. Algal Res..

[31] Ma S, Huang Y, Zhang B, Zhu X, Xia A, Zhu X, Liao Q (2023). Comprehensive modeling and predicting light transmission in microalgal biofilm. J. Environ. Manag..

[32] Ansari AA, Khoja AH, Nawar A (2017). Wastewater treatment by local microalgae strains for CO2 sequestration and biofuel production. Appl. Water Sci..

[33] Ugya AY, Ajibade FO, Hua X (2021). The efficiency of microalgae biofilm in the phycoremediation of water from River Kaduna. J. Environ. Manag..

